# Establishing a nomogram on the risk of pathological escalation of intestinal intraepithelial neoplasia in patients with colorectal intraepithelial neoplasia: a retrospective study

**DOI:** 10.3389/fmed.2025.1670165

**Published:** 2025-11-28

**Authors:** Chang Zhang, Liang Lu, Shihao Wu, Shui Jin

**Affiliations:** 1 Department of Gastroenterology, The Fourth Affiliated Hospital of Anhui Medical University, Hefei, China; 2 Department of Burns, The First Affiliated Hospital of Anhui Medical University, Hefei, China

**Keywords:** predictive model, nomogram, colorectal intraepithelial neoplasia, pathological upgrade, risk factor

## Abstract

**Objectives:**

To develop and validate a predictive model estimating the likelihood of pathological upgrading in patients with colorectal intraepithelial neoplasia (IN).

**Methods:**

Using data from 158 patients with colorectal IN confirmed by endoscopic biopsy, we employed Least Absolute Shrinkage and Selection Operator (LASSO) regression followed by multivariate logistic regression to identify key predictive factors. A nomogram was constructed based on the selected variables. The performance of these models was assessed using calibration curves, the area under the receiver operating characteristic curve (AUC), and the Hosmer-Lemeshow goodness-of-fit test. Furthermore, decision curve analysis (DCA) was utilized to evaluate the practical utility of the models, thereby exploring their potential clinical applications.

**Results:**

Four variables—rectal location, surface erosion, lesion size ≥ 30 mm, and villous histology—were incorporated into the nomogram. The model demonstrated strong discrimination (AUC = 0.822; 95% CI: 0.744–0.899) and good calibration (Hosmer–Lemeshow χ^2^ = 1.731, *p* = 0.973). Internal validation yielded a consistent AUC of 0.813. DCA confirmed the model’s broad clinical utility.

**Conclusion:**

This nomogram accurately predicts pathological upgrading in colorectal IN, allowing clinicians to identify high-risk patients early and tailor management accordingly.

## 1 Introduction

Colorectal cancer (CRC) is a malignant disease, ranking in the top three of the most common cancers in the world, ranking third in incidence and cancer-related mortality (1–3). The disease continues to pose a serious threat to global health. In China, the rapid economic development and the aging of the population have led to a continuous rise in the number of new cases of colorectal cancer and related deaths (4). The disease has become a long-standing urgent problem in the field of public health in China, highlighting the urgent need to make continuous progress in early screening, prevention strategies and treatment plans.

Early detection of colorectal cancer is crucial to improve the survival rate of patients. Research shows that when colorectal cancer is in the early stage and undergoing surgical treatment, the 5-year survival rate is close to 90% (5, 6). On the contrary, once the disease progresses to a late stage or metastasis occurs, the survival rate will drop below 10%. This sharp contrast highlights the life-saving significance of timely diagnosis and treatment. A large number of clinical studies further confirmed that the early identification and removal of precancerous lesions through colonoscopy can significantly reduce the incidence and mortality of colorectal cancer (7–10). For example, a long-term study by Zauber et al. showed that the colorectal cancer-related mortality rate of patients undergoing colonoscopic adenoma resection was reduced by 53% (7).

Colorectal cancer develops through a recognized adenoma-cancer sequence, in which intraepithelial tumor (IN) is a key precancerous stage (11–13). This concept highlights the clinical importance of accurately assessing the risk level of IN to guide timely intervention. Unlike other gastrointestinal malignant tumors such as gastric cancer, colorectal tumors limited to the mucosal layer rarely metastasize to regional lymph nodes. When complete endoscopic resection is achieved, such cases can usually be cured (14). However, once the lesion invades the submucosal layer, the treatment plan must include regional lymph node sweep. Therefore, it is very important to distinguish between high-grade intraepithelial tumor and early invasive cancer before surgery. Accurate preoperative evaluation lays the foundation for the selection of individualized treatment strategies.

At present, endoscopic forceps (EFB) are still the most reliable way to identify the characteristics of colorectal tumors. Colonoscopy plays an important role in detecting early lesions of colorectal cancer. However, its accuracy may be affected by a variety of factors, including insufficient tissue sampling depth, misjudgment caused by internal differences in the tumor, and differences in the interpretation of the results by pathologists (15–17). For this reason, the results of preoperative biopsy often do not match the final postoperative pathology report, which in turn affects the treatment decision-making. In addition, colonoscopy is an invasive and expensive detection method, which also requires intestinal preparation, which brings discomfort to patients. Therefore, it is urgent to develop simple and non-invasive follow-up tools.

Against this background, it is of great clinical significance to develop an accessible and low-cost tool to predict the potential progress of IN. Among the existing prediction methods, nomogram are particularly prominent as a practical and reliable method. By integrating multiple clinical variables and patient characteristics, it allows clinicians to estimate the probability of specific outcomes and supports more individualized decision-making (18). Although numerous predictive models have been developed for assessing colorectal cancer risk, only a limited number specifically address the pathological progression of IN (19–21).

This study was designed to explore a key question: whether a nomogram based on routine clinical indicators can accurately estimate the likelihood of pathological advancement in intestinal IN. We propose that integrating clinical, endoscopic, and pathological characteristics may yield a predictive tool with strong accuracy and meaningful clinical value.

## 2 Materials and methods

### 2.1 Patient selection

Data were collected on 158 patients diagnosed with IN through EFB at Chaohu Hospital, affiliated with Anhui Medical University, between January 2021 and January 2024. These patients subsequently underwent either EMR or ESD at our institution. The inclusion criteria for the study were defined as follows: (1) a preoperative diagnosis of LGIN or HGIN confirmed through biopsy via EFB; (2) completion of radical surgical resection with a biopsy-to-surgery interval of 6 months or less; (3) availability of comprehensive clinical and pathological data; (4) absence of any prior radiotherapy, chemotherapy, or other antitumor treatments before surgery; and (5) no history of malignancies outside the gastrointestinal tract. The exclusion criteria were: (1) abnormally elevated preoperative tumor markers, including CA19–9, CA125, or CEA; (2) inconsistent conclusions from independent pathological reviews conducted by two pathologists; and (3) a final postoperative pathological diagnosis that did not align with intraepithelial neoplasia, such as a diagnosis of inflammatory polyp, hyperplastic polyp, or invasive carcinoma.

### 2.2 Data collection

Clinical data collection was conducted using the hospital’s electronic medical record system. The data collected encompassed the following categories: (1) Patient demographics, which included gender, age, body mass index (BMI), smoking history, family history of colorectal cancer, and the presence of concomitant metabolic syndrome (MS); (2) Laboratory assessments, specifically carcinoembryonic antigen (CEA) levels measured within 1 week prior to tumor resection; (3) The quantity of biopsy specimens obtained; (4) Endoscopic characteristics, which involved the maximum tumor diameter, morphological classification as pedunculated or sessile, tumor count, anatomical location, and the presence of hyperemia/erythema, villous architecture, and ulceration. Tumor count was further categorized as either solitary or multiple, while tumor location was classified into rectum, sigmoid colon, descending colon, transverse colon, or ascending colon; (5) Categorized data regarding pathological assessment, where “pathological non-upgrade” was defined as concordant preoperative and postoperative diagnoses. Conversely, the “pathological upgrade” group was characterized by discrepancies between preoperative and postoperative diagnoses, with the latter indicating a higher-grade lesion. This specifically included two scenarios: (a) the upgrading of low-grade intraepithelial neoplasia to high-grade intraepithelial neoplasia, and (b) the transition from low-grade or high-grade intraepithelial neoplasia to a more severe pathological state.

### 2.3 Evaluation of pathological findings

All endoscopies were performed by experienced gastroenterologists (attending physician or above with at least 5 years of endoscopy experience). Performing a colonoscopy using a white-light endoscope. If abnormal areas (such as polyps or mucosal lesions) are detected, targeted biopsy sampling and further examination are performed using chromoendoscopy and magnifying endoscopy. FFPE biopsies are cut into sections, stained with H&E, and used for an initial diagnosis. To ensure the accuracy of pathology reports, senior pathologists review the diagnostic findings and make corrections when necessary. However, in cases of disagreement, the severity of the disease shall prevail.

### 2.4 Sample size estimation

The sample size was estimated using the empirical method known as events per variable (EPV) (22). Research using statistical simulations has indicated that for logistic regression, an empirical criterion of an EPV of at least 10 is recommended, meaning the number of events should be ten times the number of independent variables included to ensure robust results. The predictive model ultimately confirmed in this study comprises four predictor variables, with a total of 43 positive outcome events observed in the study cohort. Therefore, events per predictor variable is 10.75 (43/4), meeting and exceeding the empirical standard of EPV ≥ 10.

### 2.5 Statistical analysis

SPSS 24.0 and R 4.4.1 were used to carry out the statistical analysis. Statistical analysis involved comparing group percentages using the chi-square test or Fisher’s exact test. Continuous variables conforming to a normal distribution were expressed as the mean ± standard deviation, and comparisons between groups were conducted by a *t*-test; otherwise, they were expressed as the median (P25–P75), and a Mann–Whitney U test was used for intergroup comparisons. A univariate logistic regression analysis was initially employed to explore potential risk factors. Subsequently, the Lasso regression method was utilized to identify a significant combination of risk factors. Finally, a multivariate logistic regression was performed to predict pathological upgrading in colorectal IN patients, thereby constructing a predictive model and developing a nomogram. First, the Hosmer-Lemeshow test was applied to assess the model’s goodness-of-fit. Second, the ability of the model to distinguish between different outcomes was tested using the AUC. Third, a calibration curve was drawn to check how well the predicted results matched the actual outcomes. Finally, DCA was used to assess the model’s value in clinical practice. A *p*-value below 0.05 was considered to show statistical significance.

## 3 Results

### 3.1 Pathological findings following endoscopic therapy

Among the 158 patients who received endoscopic treatment, 43 showed pathological progression after the procedure, giving a progression rate of 27.2%. Of these patients, 111 were first diagnosed with LGIN based on biopsy results. Among the LGIN group, 24 cases showed progression—22 developed into HGIN, and 2 progressed to CRC, with a total progression rate of 21.6%. In addition, 47 patients were diagnosed with HGIN by biopsy, and 19 of them later developed CRC, resulting in a progression rate of 40.4%. The detailed data are shown in Table 1.

**TABLE 1:** Pathological upgrading in 158 patients with colorectal intraepithelial neoplasia following endoscopic treatment.

Biopsy pathology	n	Postoperative pathology			Pathological upgrade
		LGIN	HGIN	CRC	
LGIN	111	87 (78.4)	22 (19.8)	2 (1.8)	24 (21.6)
HGIN	47	–	28 (59.6)	19 (40.4)	19 (40.4)
Total	158	87 (55.1)	50 (31.6)	21 (13.3)	43 (27.2)

### 3.2 Baseline characteristic

After applying the inclusion and exclusion criteria, a total of 158 patients diagnosed with colorectal intraepithelial neoplasia by EFB were included in the study. The group consisted of 119 men (75.3%) and 39 women (24.7%). The median age of the patients was 57 years, with ages ranging from 24 to 91 years. Statistically significant differences were identified between the pathology-graded and ungraded groups concerning baseline clinical and pathological characteristics, including family history of colorectal cancer, number of biopsy specimens, maximum tumor diameter, tumor location, tumor hyperemia/erythema, tumor villous structure, and tumor ulceration (*P* < 0.05). Conversely, no significant differences were found between the two groups regarding gender, age, smoking history, BMI, preoperative CEA levels, presence of metabolic syndrome, pedunculated versus non-pedunculated tumors, or number of tumors (*P* > 0.05). Detailed findings are presented in Table 2.

**TABLE 2:** Comparison of basic data between the upgraded and non-upgraded groups.

Variables	Total (*n* = 158)	Non-upgraded group (*n* = 115)	Upgraded group (*n* = 43)	t/X^2^	*P*-value
Gender [n(%)]				0.979	0.323
Female	39 (24.7%)	26 (22.6%)	13 (30.2%)		
Man	119 (75.3%)	89 (77.4%)	30 (69.8%)		
Age (x ± s)	158 (58.80 ± 13.38)	115 (57.70 ± 12.39)	43 (61.74 ± 15.50)	1.699 0.357	0.130 0.550
Smoking history [n(%)]					
No	112 (70.9%)	80 (69.6%)	32 (74.4%)		
Yes	46 (29.1%)	35 (30.4%)	11 (25.6%)		
BMI [n(%)]				3.112	0.211
Normal	64 (40.5%)	48 (41.7%)	16 (37.2%)		
Underweight	73 (46.2%)	49 (42.6%)	24 (55.8%)		
Overweight	21 (13.3%)	18 (15.7%)	3 (7.0%)		
CEA [n(%)]				1.189	0.276
Negative	113 (71.5%)	85 (73.9%)	28 (65.1%)		
Positive	45 (28.5)	30 (26.1%)	15 (34.9%)		
Family history of colorectal cancer [n(%)]				5.324	0.043
No	140 (88.6%)	106 (92.2%)	34 (79.1%)		
Yes	18 (11.4%)	9 (7.8%)	11 (20.9%)		
CMS[n(%)]				0.641 20.951	0.423 < 0.001
No	89 (56.3%)	67 (58.3%)	22 (51.2%)		
Yes	69 (43.7%)	48 (41.7%)	21 (48.8%)		
Maximum tumor diameter [n(%)]					
<3 cm	126 (79.7%)	102 (88.7%)	24 (55.8%)	1.203	0.273
≥ 3 cm	32 (20.3%)	13 (11.3%)	19 (44.2%)		
Pedunculated tumor [n(%)]					
No	31 (19.6%)	25 (21.7%)	6 (14.0%)		
Yes	127 (80.4%)	90 (78.3%)	37 (86.0%)		
Number of biopsy blocks [n(%)]				5.254	0.022
1 piece	93 (58.9%)	74 (64.3%)	19 (44.2%)		
>1 piece	65 (41.1%)	41 (35.7%)	24 (55.8%)		
Congestion [n(%)]				4.132	0.042
No	94 (59.5%)	74 (64.3%)	20 (46.5%)		
Yes	64 (40.5%)	41 (35.7%)	23 (53.5%)		
Villus				18.904	< 0.001
No	122 (77.2%)	99 (86.1%)	23 (53.5%)		
Yes	36 (22.8%)	16 (13.9%)	20 (46.5%)		
Erosion [n(%)]				22.792	< 0.001
No	122 (77.2%)	100 (87.0%)	22 (51.2%)		
Yes	36 (22.8%)	15 (13.0%)	21 (48.8%)		
Number of tumor [n(%)]				0.032	0.859
Single	90 (57.0%)	66 (57.4%)	24 (55.8%)		
Multiple	68 (43.0%)	49 (42.6%)	19 (44.2%)		
Location [n(%)]				9.822	0.035
Ascending colon	29 (18.4%)	26 (22.6%)	3 (7.0%)		
Transverse colon	22 (13.9%)	17 (14.8%)	5 (11.6%)		
Descending colon	14 (8.9%)	10 (8.7%)	4 (9.3%)		
Sigmoid colon	36 (22.8%)	28 (24.3%)	8 (18.6%)		
Rectum	57 (36.1%)	34 (29.6%)	23 (53.5%)		

### 3.3 Selection of variable

#### 3.3.1 Univariate analysis of factors influencing pathological progression

Univariate analysis demonstrated that several factors, including a family history of colorectal cancer, the number of biopsy specimens, maximum tumor diameter, tumor location, presence of hyperemia, villous architecture, and ulceration, were correlated with pathological upgrade following endoscopic treatment in patients with colonic adenomatous intraepithelial neoplasia (*P* < 0.05). Conversely, variables such as sex, age, BMI, smoking history, metabolic syndrome (MS), preoperative CEA level, tumor morphology (pedunculated or sessile), and the number of tumors did not exhibit a significant association with pathological upgrade (*P* > 0.05). Comprehensive results are provided in Table 3.

**TABLE 3:** Univariate logistic regression analysis.

Variables	OR	95%CI	*P*-value
Family history of colorectal cancer	3.118	1.132–8.615	0.026
Number of biopsy blocks	2.280	1.123–4.696	0.023
Maximum tumor diameter	6.212	2.731–14.617	< 0.001
Location	5.863	1.792–26.614	0.008
Congestion	2.076	1.022–4.257	0.044
Villus	5.380	2.441–12.154	< 0.001
Erosion	6.364	2.872–14.511	< 0.001

#### 3.3.2 Selection of predictor variables using LASSO regression

**FIGURE 1:**
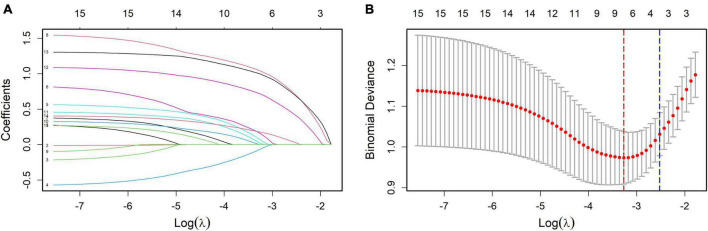
**(A)** Coefficient profiles of the variables in the LASSO regression model. Each curve represents the trajectory of a variable coefficient as the regularization parameter (λ) changes. This figure illustrates how the coefficients shrink with increasing penalty, identifying variables with the strongest predictive contributions. **(B)** Selection of the optimal penalty parameter in LASSO regression via cross-validation. The plot shows the relationship between mean cross-validated error and log(λ). The dotted lines indicate λ_min (minimum error) and λ_1se (simpler model within one standard error). λ_1se was selected as the optimal penalty for balancing model simplicity and predictive performance.

In this study, 158 patients with colorectal adenomas were included for analysis. The postoperative pathological grade was used as the dependent variable. LASSO regression was applied to identify the predictive factors, as shown in Figure 1A. The results of a 10-fold cross-validation, depicted in Figure 1B, indicated a λ_min value of 0.03815227 (log λ = −3.26617) and a λ_1se value of 0.08030687 (log λ = −2.5219). To keep the model both simple and accurate, λ_1se was chosen as the best penalty coefficient. Based on this criterion, four variables were identified as significant predictors: tumor location, villous architecture, surface erosion, and maximum tumor diameter.

#### 3.3.3 Multivariate logistic regression analysis and identification of independent risk factors

**FIGURE 2:**
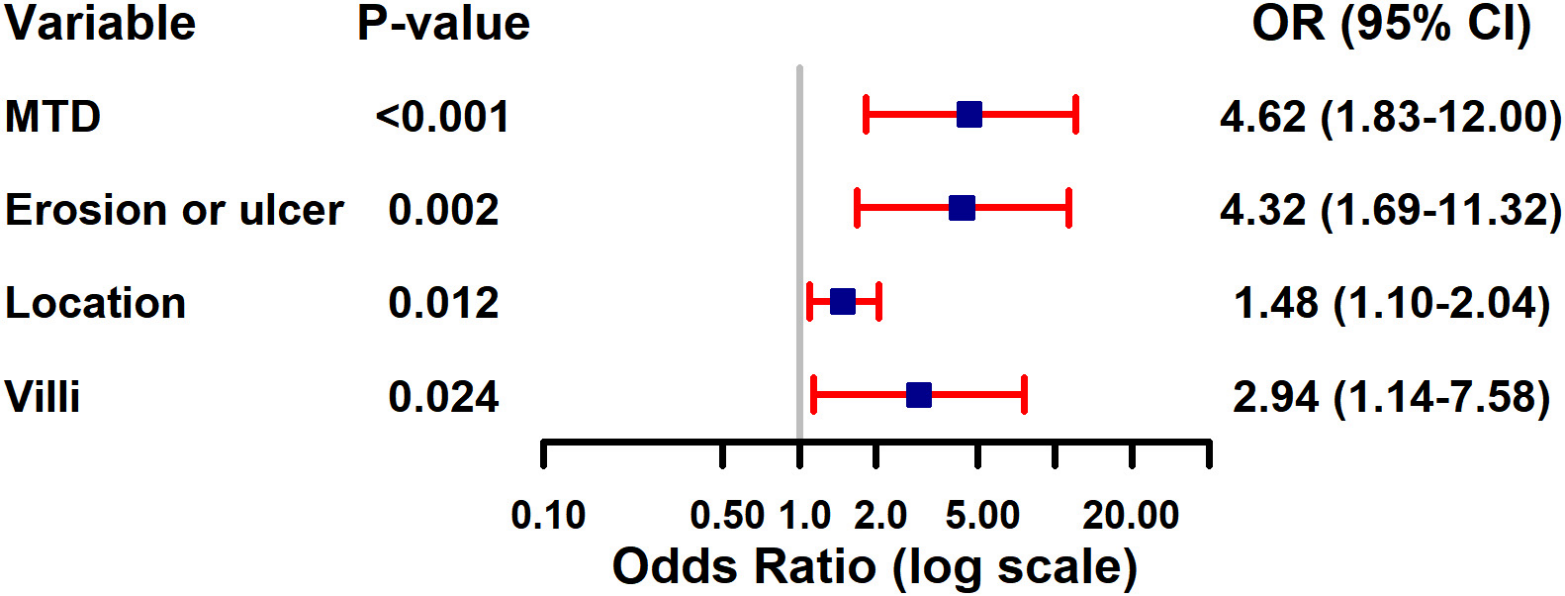
Forest plot of multivariate logistic regression analysis for risk factors of pathological upgrading in patients with colorectal intraepithelial neoplasia: - 3.521 + 1.530 × Maximum tumor diameter + 1.464 × Erosion + 0.391 × Location + 1.078 × Villus.

The four variables identified through LASSO regression were subsequently incorporated into a multivariate logistic regression model, and additional variable selection was carried out using a forward stepwise approach. The results indicated that rectal tumor location, villous architecture, surface ulceration, and a maximum tumor diameter of 30 mm or greater were independent risk factors for pathological progression following endoscopic resection of colorectal adenomas (all *P* < 0.05) (Table 4). To better visualize the magnitude of association between each factor and disease progression, a forest plot was generated based on the multivariate logistic regression model (Figure 2).

**TABLE 4:** Multivariate logistic regression analysis of factors associated with pathological upgrading.

Variables	OR	95%CI	*P*-value
Maximum tumor diameter	4.619	1.830–11.994	0.001
Erosion	4.323	1.687–11.322	0.002
Location	1.479	1.104–2.043	0.012
Villi	2.939	1.136–7.581	0.024

### 3.4 Nomogram for predicting pathologic escalation

**FIGURE 3:**
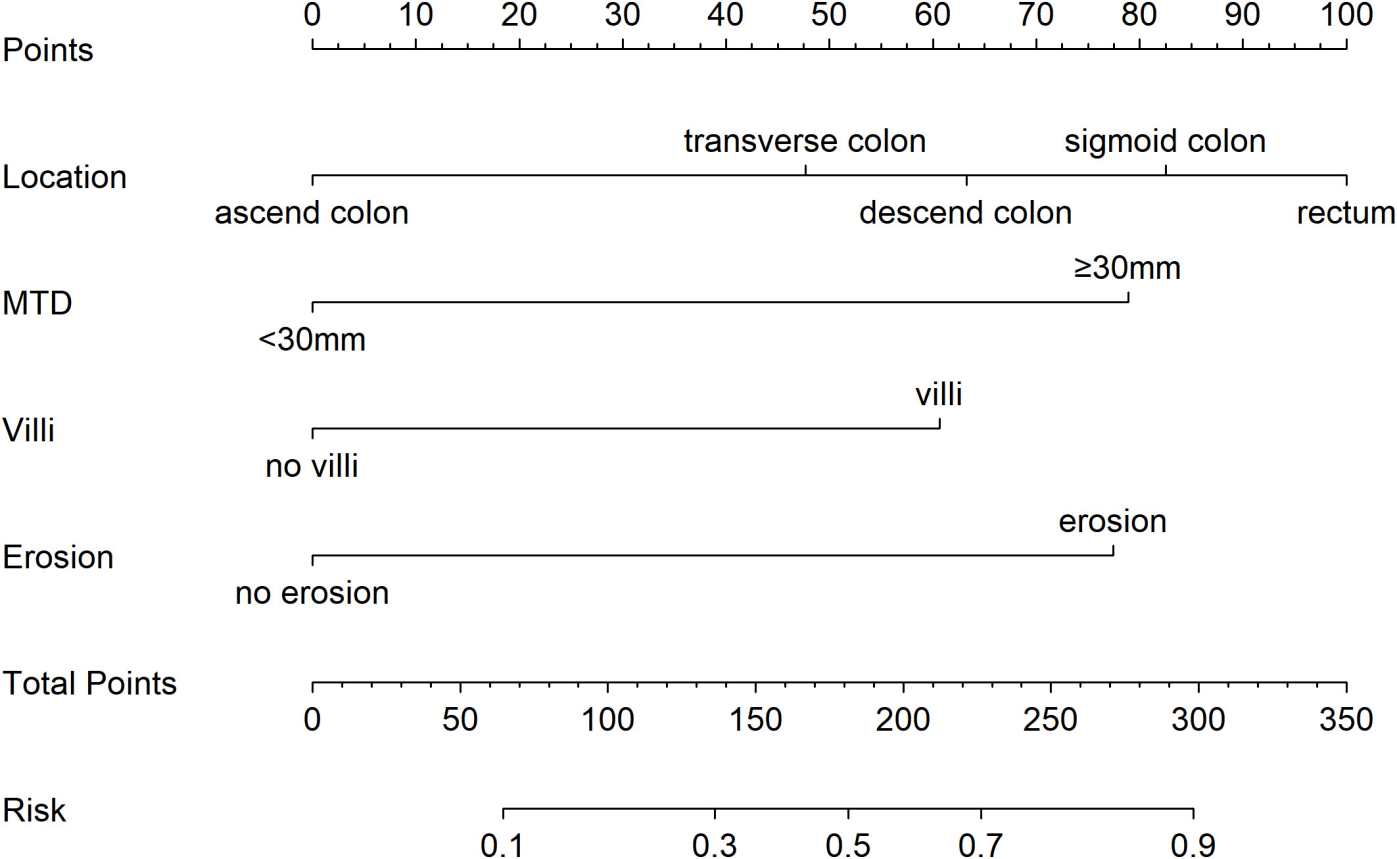
A predictive model for pathological escalation in patients with colorectal IN.

To estimate the probability of disease progression in patients with colorectal adenocarcinoma, a nomogram was developed utilizing the results from a multivariate logistic regression analysis (see Figure 3). The model incorporated four predictors: tumor location, villous architecture, surface ulceration, and a maximum tumor diameter (MTD) of 30 mm or greater. Each predictor was assigned a specific score on the upper axis of the nomogram. The individual scores were aggregated to obtain a total score, which was then projected onto the total score axis. By drawing a vertical line downward from this point to the probability axis, the estimated likelihood of pathological upgrade in patients with colorectal IN could be determined. For example, a total score of 190 means the chance of a pathological upgrade is more than 50%, while a score of 300 suggests the probability is above 90%.

### 3.5 Model validation and evaluation

**FIGURE 4:**
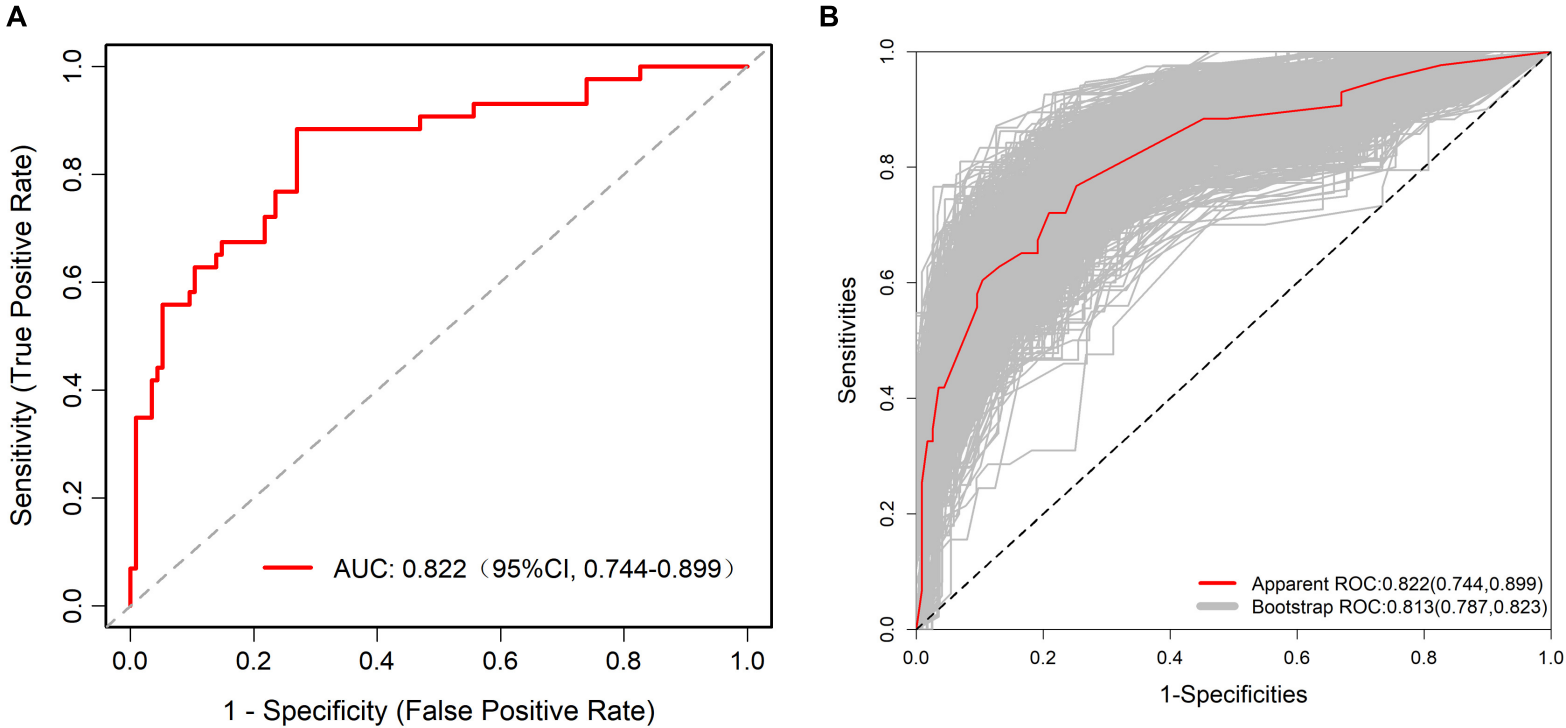
**(A)** Receiver operating characteristic (ROC) curve of the predictive model for pathological upgrading in colorectal IN patients. The model achieved an AUC of 0.822 (95% CI: 0.744–0.899), indicating good discriminative ability. **(B)** ROC curve of the internal validation of the predictive model. The internal validation yielded an AUC of 0.813 (95% CI: 0.787–0.823), confirming the model’s robustness and stability.

**FIGURE 5:**
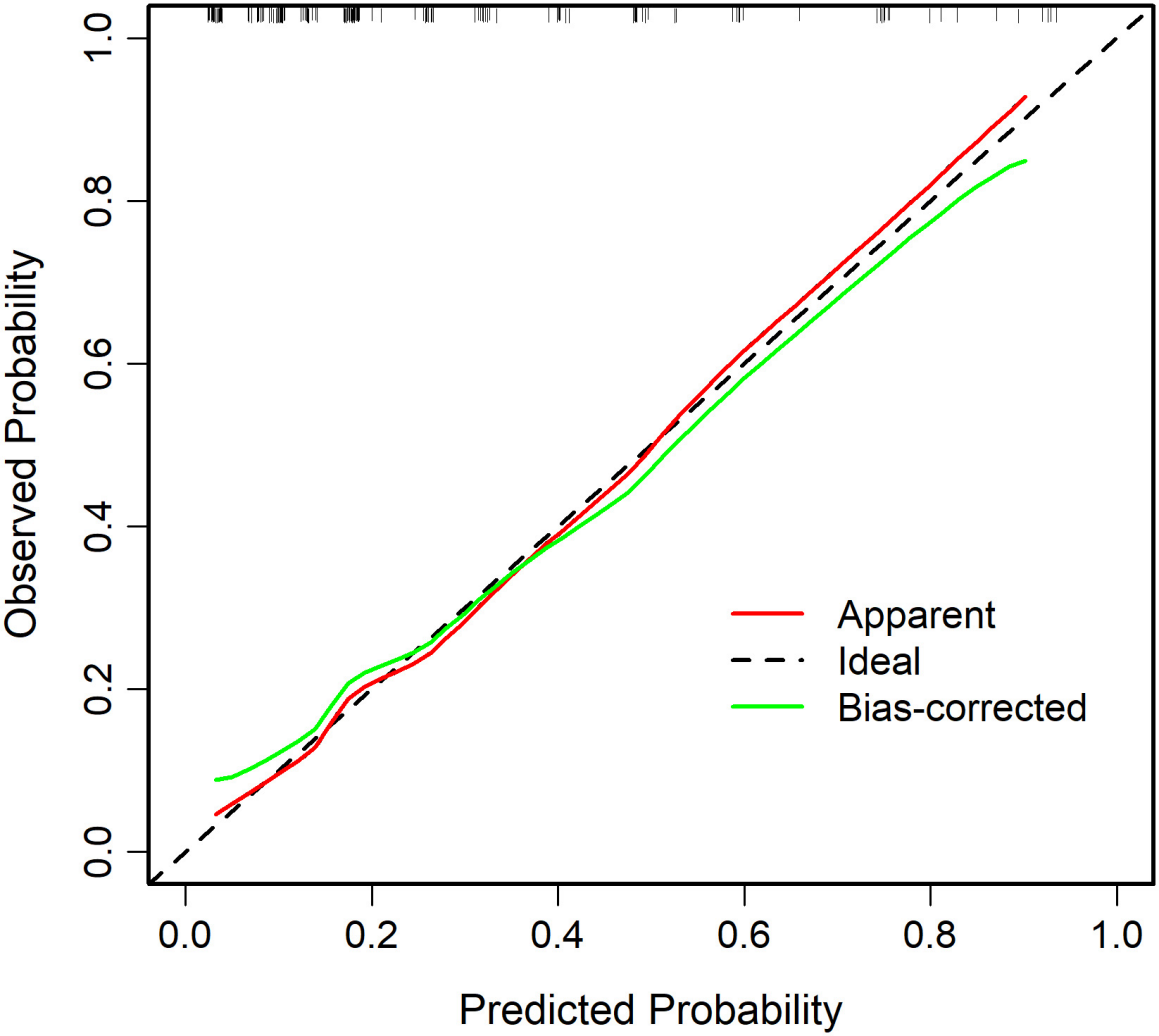
Calibration curve of the predictive model for pathological upgrading. The calibration curve shows strong agreement between predicted and observed probabilities, demonstrating good model calibration (Hosmer–Lemeshow test: χ^2^ = 1.731, *P* = 0.973).

**FIGURE 6:**
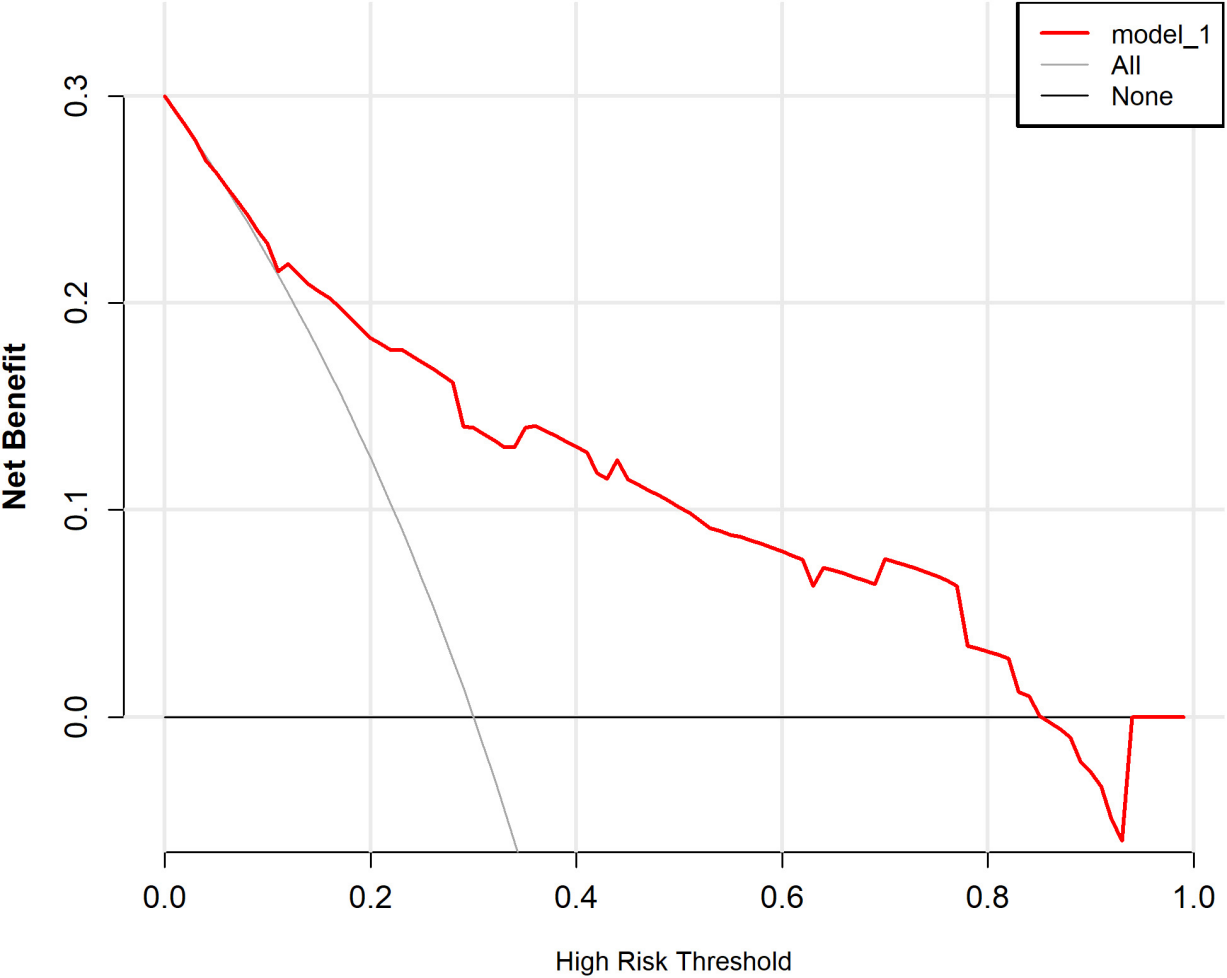
Decision curve analysis (DCA) of the predictive model for pathological upgrading. In this analysis, the horizontal axis denotes the high-risk threshold probability, whereas the vertical axis illustrates the net benefit contingent upon this threshold. Solid lines depict the net benefit associated with the predictive model, while dashed lines represent the “full treatment” and “full non-treatment” strategies, respectively. The decision curve analysis reveals that when the threshold probability ranges from 0 to 85%, the implementation of this predictive model for decision-making confers a superior net clinical benefit in comparison to both the “full intervention” and “full non-intervention” strategies. This finding underscores the substantial clinical utility of the model.

The ROC curve analysis showed that the predictive model reached an AUC of 0.822, with a 95% CI of 0.744–0.899, for predicting pathological upgrading in patients with colorectal intraepithelial neoplasia (see Figure 4A). The model had a sensitivity of 76.74% and a specificity of 74.78%. Internal validation produced an AUC of 0.813 (95% CI: 0.787–0.823) (see Figure 4B). In the calibration curve, the x-axis shows the predicted probability of pathological upgrading, and the y-axis shows the actual observed probability (see Figure 5). The diagonal line represents an ideal situation where the predicted and observed values are the same. The model is considered accurate when the calibration curve is close to this diagonal line. The Hosmer–Lemeshow test gave a chi-square value of 1.731 and a *P*-value of 0.973 (*P* > 0.05), which means the model had good fit and reliable predictions. To assess clinical usefulness, DCA was used to estimate the net benefit of using the nomogram to predict pathological progression in patients with colorectal intraepithelial neoplasia (see Figure 6).

## 4 Discussion

Colorectal cancer (CRC) is a common malignant tumor of the digestive system, marked by high rates of illness and death. The outcome of CRC is closely related to how early it is diagnosed and treated. Therefore, finding CRC at an early stage is very important, as it allows timely treatment and greatly improves both survival and prognosis. IN is an important precancerous stage in the development of CRC. Accurate risk assessment of IN is a key part of clinical decision-making, as it helps determine whether a patient should undergo endoscopic removal or surgery. However, current preoperative diagnoses based on EFB often differ from final postoperative pathology results. These differences usually occur because of sampling mistakes and variations in how pathologists interpret tissue samples. Thus, there is an urgent clinical need to create a dependable tool that can predict the risk of pathological progression of IN before surgery. Such a tool would help doctors design more personalized and effective treatment plans for patients.

In order to solve this clinical dilemma, we have developed a nomogram to provide doctors with a preoperative risk assessment tool. The clinical application of the nomogram can be implemented through the following steps: first, according to the clinical characteristics of the patient, such as lesion size, morphological characteristics, pathological grade, etc., the score is determined on the corresponding variable axis; second, the scores of each variable are summed to obtain the total score; finally, the corresponding risk probability of pathological upgrading is read on the total score axis. For example, a patient with a large lesion size, specific morphological characteristics, and high-risk pathological classification may have a total score corresponding to a 65% risk of progression, suggesting that surgical treatment should be considered. This intuitive tool enables doctors to conduct individualized risk assessment before surgery, providing a quantitative basis for formulating accurate treatment plans and thus optimizing the clinical decision-making process.

The LASSO method was more effective than the stepwise selection approach in handling multicollinearity and reducing overfitting in the regression model. This study first uses univariate analysis to screen variables, and then selects variables through LASSO regression and multivariate logical regression. In the end, four variables with the strongest predictive value were retained: tumor size (≥≥ 30 mm), villi structure, ulcer and rectal site. The model based on these four prediction factors shows strong identification ability and reliable calibration. Overall, these findings have important clinical value, indicating that the model can be used as a practical tool for identifying patients at high risk of disease progression.

This study analyzed 158 patients with colorectal intraepithelial tumor, of which 43 cases were pathologically upgraded, with an overall progression rate of 27.2%. Among the patients who were first diagnosed with LGIN, 24 cases (21.6%) showed progress; while among the patients with HGIN, 19 cases (40.4%) had further deterioration. These observations highlight the malignant potential of HGIN. As a key stage of the adenoma-cancer sequence, the molecular and genetic changes of HGIN often foreshadow the early initiation of malignant lesions. Once such lesions are confirmed, regardless of the endoscopic performance, they should be regarded as “cancerous cancer that is about to worsen” and should be removed in time through surgery. In contrast, although the overall upgrade rate of LGIN is low, its progress is not random. If the lesion is accompanied by high-risk characteristics such as surface erosion, large diameter or located in the rectum, the risk of upgrading will increase significantly. Therefore, clinicians should attach the same importance to LGIN lesions that conform to the above characteristics under endoscopy, rather than underestimating the risk based on baseline pathological grading alone. This discovery helps to accurately identify high-risk individuals in heterogeneous LGIN populations, thus optimizing clinical treatment decisions.

Many studies have shown that EFB alone has limited diagnostic accuracy and often fails to reflect the true histological type of the lesion. For lesions measuring 10–20 mm and 20–40 mm, the mismatch rates between biopsy results and final pathology were 35 and 37%, respectively. A small study by Pugliese et al. (23) also found that differences between EFB and postoperative pathology increased as lesion size grew. Therefore, for larger lesions, endoscopists should be more cautious about the possibility of pathological upgrading after surgery and recognize the limited reliability of EFB results. In line with previous research, this study found that a MTD of 30 mm or more was an independent risk factor for higher grades of intraepithelial neoplasia. Adenocarcinomas usually have mixed histological structures (24). As lesions become larger, the chance of obtaining enough cancerous tissue through forceps biopsy decreases, which reduces the accuracy of EFB samples. Thus, larger tumors are more likely to produce unrepresentative biopsy specimens. In addition, the risk of malignant transformation also increases with tumor size, which may further explain why larger lesions are linked to higher pathological grades.

Early studies have shown that adenomas with villous components, especially those containing more than 25% villous tissue, carry a much higher risk of progressing to invasive carcinoma or high-grade dysplasia after surgery compared with other types of adenomas (25). Villous structures are often underestimated in biopsy samples because of sampling errors. They also show stronger biological activity and higher cancer potential. Therefore, complete removal and close follow-up of these lesions are strongly recommended. At the molecular level, villous adenomas share more features with carcinomas. They often contain driver mutations such as APC and show genomic instability, such as a high homologous recombination deficiency (HRD) score, which supports their tendency for fast malignant change (26). In addition, about 60% of villous adenomas express vascular endothelial growth factor (VEGF), which may promote blood vessel growth and tumor spread (27). Studies have also found that villous adenomas have the lowest 5-year cancer-specific survival rate (74.1%), compared with 84.2% for tubular adenocarcinoma and 81.5% for tubulovillous adenocarcinoma, suggesting a stronger tendency toward malignant transformation (28).

In endoscopic operations, it is very important to accurately identify and completely remove colorectal lesions accompanied by erosion or ulcers. Theoretically, the erosion area is more prone to malignancy due to hypoxic microenvironment (HIF-1α↑, p53 mutation), and the surface necrotic tissue is prone to biopsy errors (29). Studies have shown that there is a link between colorectal cancer and the occurrence of tumor erosion ulcers, or even an independent link (30, 31). The research of foreign scholar Hong Junbo found that the escalation rate of tumor erosion is as high as 42.6%, and the probability of pathological escalation of tumor erosion is 7.12 times that of non-erosion (OR = 7.12; 95% CI, 3.91–12.94; *P* < 0.001) (32). For high-risk erosion lesions, accurate identification and thorough resection under endoscopy is the core link to prevent pathological escalation, avoid follow-up risks and guide correct clinical decision-making.

From an anatomical point of view, the rectum and sigmoid colon are more prone to chronic inflammation, and long-term exposure to fecal carcinogens (33). These areas repeatedly undergo the cycle of mucosal damage, regeneration and repair, which increases the risk of DNA replication errors, which leads to genetic mutations that promote tumor development (34, 35). In addition, the abundant blood vessels and lymphatic networks in this area enable the accumulation of inflammatory mediators and growth factors, forming a microenvironment conducive to tumor transformation. The size of the tumor (especially the maximum diameter) is also recognized as an important determinant of malignant progression. The research of Hong Junbo and others shows that the rectum is an independent risk factor for the atypical progression of appendicitis, and the risk of abnormal progression of distal adenoma is 3.29 times higher than that of proximal lesions (*P* < 0.01) (32). Consistent with the above findings, our research results show that the risk of malignant transformation of rectal adenomas is 1.48 times higher than that of adenomas in other parts.

This research has made several notable contributions to this field. It proposes a new predictive model aimed at assessing the possibility of pathological progression in patients with IN. The model serves as a practical tool that may assist clinicians in identifying high-risk individuals early, thereby improving intervention timing and potentially reducing mortality associated with colorectal cancer. Unlike most existing models that are mainly dedicated to predicting the risk of colorectal cancer, this study focusses on the transition stage represented by IN. By targeting this intermediate stage, the model fills an important gap in the current risk stratification system. Another advantage of the model is that it only relies on conventional clinical variables, so that its explanation is concise and suitable for daily clinical practice. The effectiveness of the model was evaluated by calibration curve, ROC analysis and decision curve analysis. The results showed that the model showed strong clinical practicality, satisfactory accuracy and reliable identification performance in different risk categories.

There are some significant limitations in this study. First of all, it adopts a single-center retrospective analysis design, which may introduce the risk of selection bias and measurement bias. Although the single-center design has advantages, such as maintaining the consistency of the patient’s demographic characteristics, endoscopic operation and diagnostic standards, so as to ensure that the data is more uniform. However, this feature also limits the universality of the model, and it is difficult to promote it to other hospitals, populations or clinical environments. Another limitation lies in the retrospective characteristics of the data itself. Because the research relies on existing medical records, some variables are missing or observer bias, which may affect the overall reliability of the model. Although techniques such as LASSO regression and internal self-lifting verification are used to minimize the fit, the model has not been evaluated by an independent external data set. Therefore, additional verification through large-scale multi-center prospective research is crucial to confirm its wide applicability and predictive robustness.

Looking forward to the future, several directions can further strengthen this research. The next stage will focus on multi-center prospective research to realize the external verification of the model. By collecting different levels of hospital and cross-regional data, we aim to rigorously evaluate the real generalization ability of the model. At the same time, we will focus on developing online prediction tools or integrating the model into the hospital’s electronic health record system as an integral part of the clinical decision-making support platform. Such integration will realize the real-world performance verification of the model, assist clinicians to more accurately assess preoperative risks and formulate personalized treatment plans. In addition, the use of prospective research design can realize structured postoperative follow-up, so as to evaluate the impact of the model on the long-term prognosis and the overall course of the disease. This method is expected to eventually bridge the gap between predictive modeling and clinical practice application.

## 5 Conclusion

This study analyzed a number of clinical pathological data of patients with colorectal endoepithelial tumor, including the number of biopsy specimens, laboratory indicators, endoscopic characteristics and basic demographic information. Based on these variables, a prediction model is constructed to evaluate the probability of pathological progression. The study identified four independent risk factors: tumor site, mucosal erosion, focal diameter ≥ 30 mm, and villi histological composition. Based on these predictive indicators, we have developed predictive charts to assist clinicians in assessing the risk of disease progression more accurately. This visualization tool can realize the early identification of high-risk patients, provide a basis for the selection of appropriate treatment plans, and help improve the effectiveness of surgery. By guiding timely intervention, it may also help decrease the overall incidence of colorectal cancer and related mortality.

## Data Availability

The original contributions presented in the study are included in this article/supplementary material, further inquiries can be directed to this corresponding author.
